# Survey for Incidence of Cancer as a Measure of Outcome in the JACC Study

**DOI:** 10.2188/jea.15.S80

**Published:** 2005-05-16

**Authors:** Mitsuru Mori, Fumio Sakauchi, Masakazu Washio, Kotaro Ozasa, Yoshiyuki Watanabe, Takesumi Yoshimura, Akiko Tamakoshi

**Affiliations:** 1 Department of Public Health, Sapporo Medical University School of Medicine.; 2 Department of Epidemiology for Community Health and Medicine, Kyoto Prefectural University of Medicine Graduate School of Medical Science.; 3 Department of Clinical Epidemiology, Institute of Industrial Ecological Sciences, University of Occupational and Environmental Health.; 4 Department of Preventive Medicine/Biostatistics and Medical Decision Making, Nagoya University Graduate School of Medicine.

**Keywords:** cohort studies, incidence, cancer

## Abstract

BACKGROUND: As endpoint of cohort studies on cancer, the incidence, rather than the mortality is preferable. Of 45 areas in the Japan Collaborative Cohort Study (JACC Study), surveys for incidence of cancer were conducted in 24.

METHODS: The proportion of the study subjects aged 40 to 79 years in areas of survey for the incidence of cancer (65,184 persons) was 58.2% of the total subjects of the JACC Study (110,792 persons). Among the 24 areas of survey for the incidence of cancer (ASI), 10 areas were combined because of similarity. Then, we present the incidence rate of cancer among 15 ASI unified from the 24 ASI by gender according to area. We also report the completeness of the survey for cancer incidence presenting the mortality-incidence ratio (MI ratio) among the ASI.

RESULTS: Where a population-based cancer registry was utilized, the MI ratio deviated from 0.31 to 0.61 in the male subjects and from 0.15 to 0.53 in the female subjects. However, where a population-based cancer registry was not used, the MI ratio deviated from 0.25 to 0.72 in the male subjects and from 0.13 to 0.79 in the female subjects, and there was an area where the MI ratio exceeded 0.70 in both of the male and female subjects.

CONCLUSION: Establishment of population-based cancer registries is strongly desired throughout Japan to assess risk factors of cancer development for primary prevention of cancer.

As endpoint of cohort studies on cancer, the incidence, rather than the mortality is preferable, if risk factors have been assessed with regard to cancer development. Many cohort studies make use of existing routine surveillance systems of the incidence of cancer, such as population-based cancer registries, to ascertain the outcome of interest.^1^ Of 45 cities, towns, and villages that participated in the Japan Collaborative Cohort Study (JACC Study) for Evaluation of Cancer Risk sponsored by the Ministry of Education, Science, Sports and Culture of Japan (Monbusho),^2^ the incidence of cancer was measured in 24 areas from the Hokkaido area to the Kyushu area, and we called these the areas of survey for the incidence (ASI). Because the established population-based cancer registries did not cover all of Japan,^3^ some of the ASI did not utilize these registries for identifying subjects with cancer. Where established population-based cancer registries were not available, hospital-based cancer registries or inpatient records of the hospitals treating cancer patients were used for identifying the subjects with cancer.

We report here the number of the subjects with cancer as well as the completeness of the survey for cancer incidence^4^ presenting the mortality-incidence ratio by gender according to area among the ASI. We not only show the crude rates and the age-adjusted rates of cancer incidence, but those of cancer mortality by gender according to area among the ASI.

Our entire study design, which comprised singular and collective use of epidemiologic data and biological materials (serum only), was approved in 2000 by the Ethical Board at Nagoya University School of Medicine, where the central secretariat of the JACC study is located.

## Areas and tools of survey for incidence of cancer

Of 45 areas in the JACC Study, surveys for incidence of cancer were conducted in 24. Among them, 10 areas were located in the same prefecture in Kyushu, and were surveyed by the same institution, utilizing a population-based cancer registry. Consequently, we combined these 10 areas, and present the data of 15 ASI unified from the 24 ASI by gender according to area in Table 1. The proportion of the study subjects aged 40 to 79 years in the ASI (65,184 persons) was 58.8% among them in the JACC Study (110,792 persons).

**Table 1.** Tools of survey for cancer incidence and number of subjects aged 40 to 79 years at the baseline survey.

Temporary number of areas of survey*	Tools of survey for cancer incidence	Tentative end of observation (year) for cancer incidence	No. of male subjects	No. of female subjects	Total no. of subjects
P1	Population-based cancer registry	1994	3,113	7,162	10,275
P2	Population-based cancer registry	1997	1,145	4,071	5,216
P3	Population-based cancer registry	1997	1,981	2,432	4,413
P4	Population-based cancer registry	1997	1,719	2,564	4,283
P5	Population-based cancer registry	1997	1,226	1,405	2,631
P6	Population-based cancer registry	1997	979	1,058	2,037
P7	Population-based cancer registry	1997	987	1,036	2,023
P8	Population-based cancer registry	1997	773	901	1,674
P9	Population-based cancer registry	1997	749	847	1,596
P10	Population-based cancer registry	1997	624	971	1,595
P11	Population-based cancer registry	1997	363	542	905
O1	Others	1997	10,631	12,317	22,948
O2	Others	1997	1,142	1,840	2,982
O3	Others	1997	784	1,169	1,953
O4	Others	1997	248	405	653

Total			26,464	38,720	65,184

* : Population-based cancer registries were utilized for areas from P1 to P11, but hospital-based cancer registries or inpatients’ records of hospitals treating cancer patients were used for areas from O1 to O4.

As tools to survey for cancer incidence, population-based cancer registries were utilized in 11 out of the 15 ASI, and hospital-based cancer registries or inpatients’ records of hospitals treating cancer patients were used in the remaining 4 ASI. Although the tentative end of observation was in 1997 in 14 of the 15 ASI for this article, it was in 1994 in an area because of incidental interruption in the survey for cancer incidence, as shown in Table 1. Table 1 also shows the number of subjects, aged 40 to 79 years at the baseline survey.

## Incidence of cancer and completeness of survey

Excluding the subjects who had cancer before the baseline survey, we present the numbers of the study subjects, the dead subjects, the subjects dead who died from cancer, and the subjects with incident cancer during the periods of observation by sex according to area in Table 2. The proportions of cancer deaths among total deaths varied from 22.4% to 54.7% among the male subjects, and from 14.3% to 71.4% among the female subjects as shown in Table 2.

**Table 2.** Number of subjects with cancer and mortality/incidence ratio for cancer incidence by gender according to area, aged 40 to 79 years at the baseline survey, excluding subjects who had cancer before the baseline survey.

Male							Female					
	
Temporary number of areas of survey*	No. of subjects	No. of dead subjects (1)	No. of subjects dead due to cancer (2)	No. of subjects with incident cancer (3)	Percent of cancer deaths in total deaths {(2)/(1)x100}	Mortality/ incidence (MI) ratio {(2)/(3)}	No. of subjects	No. of dead subjects (1)	No. of subjects dead due to cancer (2)	No. of subjects with incident cancer (3)	Percent of cancer deaths in total deaths {(2)/(1)x100}	Mortality/ incidence (MI) ratio {(2)/(3)}
P1	2,842	208	87	240	41.8	0.36	6,512	172	58	251	33.7	0.23
P2	1,131	53	29	58	54.7	0.50	3,990	68	44	128	64.7	0.34
P3	1,920	262	67	144	25.6	0.47	2,361	158	39	104	24.7	0.38
P4	1,674	288	109	206	37.8	0.53	2,503	172	67	127	39.0	0.53
P5	1,150	76	17	77	22.4	0.22	1,358	43	10	37	23.3	0.27
P6	974	107	40	81	37.4	0.49	1,042	50	18	45	36.0	0.40
P7	972	89	25	53	28.1	0.47	1,081	62	14	34	22.6	0.41
P8	755	105	33	84	31.4	0.39	891	70	17	43	24.3	0.40
P9	733	115	46	76	40.0	0.61	835	46	11	23	23.9	0.48
P10	615	19	8	26	42.1	0.31	954	7	5	34	71.4	0.15
P11	361	26	8	20	30.8	0.40	530	15	5	11	33.3	0.45
O1	10,524	1,137	390	992	34.3	0.39	12,136	775	266	682	34.3	0.39
O2	1,136	106	38	58	35.8	0.66	1,797	69	25	38	36.2	0.66
O3	782	123	50	69	40.7	0.72	1,156	83	34	43	41.0	0.79
O4	245	11	3	12	27.3	0.25	397	14	2	15	14.3	0.13

Total	25,814	2,725	950	2,196	34.9	0.43	37,543	1,804	615	1,615	34.1	0.38

* : Population-based cancer registries were utilized for areas from P1 to P11, but hospital-based cancer registries or inpatients’ records of hospitals treating cancer patients were used for areas from O1 to O4.

Completeness of survey for incidence of cancer is defined as the extent to which all the incident cancers occurring in a target population are included for the study subjects.^4^^,^^5^ Ideally, completeness should be close to 100%, so that comparison of rates among areas will reflects true differences in the risk of cancer and not artifacts of the survey process. Generally, however, most ASI would have the possibility of incompleteness due to failure to identify and include incident cancers. Completeness is assessed by measuring the percentage of the subjects with cancer identified from the death certificate only (DCO%), the percentage of the subjects with cancer first notified via death certificate (DCN%), and the mortality/incidence ratio (MI ratio). Although the DCO% and the DCN% could not be estimated because of lack in relevant information, the MI ratio was evaluated as shown in Table 2.

If the MI ratio exceeds 0.70, it is usually a signal of under-registration^5^ of incident cancers. As shown in Table 2, where a population-based cancer registry was utilized, the MI ratio deviated from 0.31 to 0.61 in the male subjects and from 0.15 to 0.53 in the female subjects. However, where a population-based cancer registry was not used, the MI ratio deviated from 0.25 to 0.72 in the male subjects and from 0.13 to 0.79 in the female subjects, and there was an area where the MI ratio exceeded 0.70 in both of the male and female subjects.

## Crude and age-adjusted rates of incidence and mortality of cancer

Cancer incidence varies strongly with age. Hence, standardization of age increases the ability to compare rates of cancer among different areas by distribution of age.^6^ Table 3 shows the average age in years at the baseline survey, person-years (PYs) of observation, the crude and age-adjusted rates of cancer incidence per 100,000 PYs, and the crude and age-adjusted rates of cancer mortality per 100,000 PYs by gender according to area. The age-adjusted rates were calculated for the subjects aged 40 to 79 years with the direct methods^7^ standardized to the 1985-model Japanese population.^8^ The age-adjusted rates of cancer incidence varied from 344.14 to 1187.97 in the male subjects and from 216.83 to 597.00 in the female subjects. Likewise, the age-adjusted rates of cancer mortality varied from 95.66 to 607.64 in the male subjects and from 33.89 to 265.55 in the female subjects.

**Table 3.** Crude and age-adjusted rates of incidence and mortality of cancer by gender according to area among subjects aged 40 to 79 years at the baseline survey, excluding subjects who had cancer before the baseline survey.

Male							Female					
	
Temporary number of areas of survey*	Average age (years) at baseline survey	Person-years (PYs)	Crude rate of cancer incidence per 100,000 PYs	Age-adjusted rate of cancer incidence per 100,000 PYs	Crude rate of cancer mortality per 100,000 PYs	Age-adjusted rate of cancer mortality per 100,000 PYs	Average age (years) at baseline survey	Person-years (PYs)	Crude rate of cancer incidence per 100,000 PYs	Age-adjusted rate of cancer incidence per 100,000 PYs	Crude rate of cancer mortality per 100,000 PYs	Age-adjusted rate of cancer mortality per 100,000 PYs
P1	63.6	14192.71	1691.01	1069.00	634.13	334.84	62.9	32917.20	762.52	650.61	188.35	117.52
P2	55.4	8616.74	661.50	777.54	336.55	340.60	53.2	30660.35	417.48	451.92	143.51	172.57
P3	58.7	15311.94	940.44	777.90	450.63	353.26	59.2	19501.30	533.30	444.95	199.99	151.66
P4	59.5	14060.98	1465.05	1187.97	782.31	607.64	58.6	22219.82	571.56	507.02	301.53	265.55
P5	54.8	7994.89	963.12	855.39	212.64	197.08	57.0	9775.74	378.49	282.29	102.29	77.67
P6	57.5	7544.42	1073.64	957.60	530.19	456.93	58.2	8284.01	543.22	474.65	217.29	181.29
P7	54.0	7360.49	720.06	818.16	339.65	386.05	55.6	7907.96	429.95	433.77	177.04	178.94
P8	59.5	6583.27	1275.96	984.08	516.46	372.20	58.7	8045.27	534.48	408.89	223.73	174.09
P9	63.3	5472.14	1388.85	789.90	840.62	479.53	63.6	5875.20	391.48	216.83	187.23	98.44
P10	49.7	4987.53	521.30	344.14	160.40	101.36	50.2	7786.30	436.66	261.81	64.22	33.89
P11	53.4	2668.59	749.46	780.05	299.78	313.47	54.0	4158.64	264.51	303.14	120.23	152.06
O1	56.7	86420.24	1147.88	1068.14	451.28	415.87	57.7	102911.06	662.71	597.00	258.48	218.80
O2	59.0	9010.71	643.68	486.19	421.72	322.37	58.0	14613.08	260.04	243.67	171.08	165.93
O3	60.2	6995.65	986.33	792.76	714.73	546.09	59.2	10707.43	401.59	316.47	317.54	244.77
O4	55.1	874.53	640.16	515.53	160.04	95.66	54.5	2972.76	504.58	411.80	67.28	50.51

* : Population-based cancer registries were utilized for areas from P1 to P11, but hospital-based cancer registries or inpatients’ records of hospitals treating cancer patients were used for areas from O1 to O4.

For comparison to the whole Japanese population, we calculated the age-adjusted rate of cancer incidence with reference to the data for age-specific rates of cancer incidence for those aged 40 to 79 years in 1993 in Japan,^9^ standardized to the 1985-model Japanese population,^8^ as well. As a result, the age-adjusted rates of cancer incidence were 751.21 and 429.77 per 100,000 in Japanese males and females, respectively. Consequently, the medians of the age-adjusted rate of cancer incidence in the males (792.76 per 100,000 PYs) and the females (411.80 per 100,000 PYs) of the 15 ASI were very much close to the above figure.

Similarly, for further comparison to the whole Japanese population, we calculated the age-adjusted rate of cancer mortality with reference to the data on age-specific rates of cancer mortality in persons aged 40 to 79 years in 1995 in Japan,^10^ standardized to the 1985-model Japanese population,^6^ as well. As a result, the age-adjusted rates of cancer mortality were 436.08 and 204.42 per 100,000 in Japanese males and females, respectively. Accordingly, the medians of the age-adjusted rate of cancer mortality in the males (353.26 per 100,000 PYs) and the females (165.93 per 100,000 PYs) of the 15 ASI were slightly lower than the above figure. Because the study subjects were not only selected from the participants of health check-up program in some areas, but also they were probably liable to examine their symptom at medical institution, earlier detection and treatment might cause relatively higher rate of cancer incidence and lower rate of cancer mortality among them.

## Conclusion

Because death registries are available in the whole areas of the JACC Study, most of the previous reports of the JACC Study made use of the cancer mortality for the outcome. However, it is well known that mortality rates depend on prognosis or survival.^6^ That is, mortality rates do not meaningfully reflect incidence rates, especially, in cancers with a favorable prognosis like skin, thyroid, breast, and prostate cancer.

Incidence rates provide the clearest measure of the burden of carcinogenic exposures at the population level.^6^ However, only 58.2% of the study subjects were involved in the ASI of the JACC Study, and considerable variations were observed in the MI ratio of the survey for incidence. Therefore, the findings from the results of analysis of the subjects with incident cancer should be reported with the MI ratio of Table 2. Furthermore, establishment of population-based cancer registries is strongly desired throughout Japan to assess risk factors of cancer development for primary prevention of cancer.

## MEMBER LIST OF THE JACC STUDY GROUP

The present investigators involved, with the co-authorship of this paper, in the JACC Study and their affiliations are as follows: Dr. Akiko Tamakoshi (present chairman of the study group), Nagoya University Graduate School of Medicine; Dr. Mitsuru Mori, Sapporo Medical University School of Medicine; Dr. Yutaka Motohashi, Akita University School of Medicine; Dr. Ichiro Tsuji, Tohoku University Graduate School of Medicine; Dr. Yosikazu Nakamura, Jichi Medical School; Dr. Hiroyasu Iso, Institute of Community Medicine, University of Tsukuba; Dr. Haruo Mikami, Chiba Cancer Center; Dr. Yutaka Inaba, Juntendo University School of Medicine; Dr. Yoshiharu Hoshiyama, Showa University School of Medicine; Dr. Hiroshi Suzuki, Niigata University School of Medicine; Dr. Hiroyuki Shimizu, Gifu University School of Medicine; Dr. Hideaki Toyoshima, Nagoya University Graduate School of Medicine; Dr. Shinkan Tokudome, Nagoya City University Graduate School of Medical Science; Dr. Yoshinori Ito, Fujita Health University School of Health Sciences; Dr. Shuji Hashimoto, Fujita Health University School of Medicine; Dr. Shogo Kikuchi, Aichi Medical University School of Medicine; Dr. Akio Koizumi, Graduate School of Medicine and Faculty of Medicine, Kyoto University; Dr. Takashi Kawamura, Kyoto University Center for Student Health; Dr. Yoshiyuki Watanabe, Kyoto Prefectural University of Medicine Graduate School of Medical Science; Dr. Tsuneharu Miki, Kyoto Prefectural University of Medicine Graduate School of Medical Science; Dr. Chigusa Date, Faculty of Human Environmental Sciences, Mukogawa Women’s University ; Dr. Kiyomi Sakata, Wakayama Medical University; Dr. Takayuki Nose, Tottori University Faculty of Medicine; Dr. Norihiko Hayakawa, Research Institute for Radiation Biology and Medicine, Hiroshima University; Dr. Takesumi Yoshimura, Institute of Industrial Ecological Sciences, University of Occupational and Environmental Health, Japan; Dr. Akira Shibata, Kurume University School of Medicine; Dr. Naoyuki Okamoto, Kanagawa Cancer Center; Dr. Hideo Shio, Moriyama Municipal Hospital; Dr. Yoshiyuki Ohno, Asahi Rosai Hospital; Dr. Tomoyuki Kitagawa, Cancer Institute of the Japanese Foundation for Cancer Research; Dr. Toshio Kuroki, Gifu University; and Dr. Kazuo Tajima, Aichi Cancer Center Research Institute.
